# The why, how, and what of indicator-based monitoring of nature-based solutions: Perspectives from EU and LAC city practitioners

**DOI:** 10.1007/s13280-025-02174-0

**Published:** 2025-04-22

**Authors:** Martina van Lierop, Cynnamon Dobbs, Alexander van der Jagt, Andrea Skiba, Camila Flores, Giuliano Maselli Locosselli, Denise Duarte, Aude Zingraff-Hamed, Stephan Pauleit

**Affiliations:** 1 Chair for Strategic Landscape Planning and Management, School of Life Sciences Weihenstephan, Technical University of Munich, Emil-Ramann-Straße 6, 85354 Freising, Germany; 2 Centro de Estudios Territoriales, Universidad de los Andes Chile, Monsenor Alvaro del Portillo 12455, Las Condes, Santiago Chile, Chile; 3 Institute for Place, Environment and Society, School of Energy, Geoscience, Infrastructure and Society, Heriot-Watt University, William Arrol Building, Edinburgh, EH14 4AS UK; 4 Center for Nuclear Energy in Agriculture, University of São Paulo, Av. Centenário, 303 - São Dimas, Piracicaba, SP 13416-000 Brazil; 5 Faculty of Architecture, Urbanism and Design, University of São Paulo, Rua do Lago 876 Cidade Universitária, São Paulo, SP 05508-080 Brazil; 6 ENGEES National Institute for Water and Environmental Engineering, 1 cour des cigarières, 61039, 67070 Strasbourg, France; 7 CNRS, ENGEES, UMR 7362 LIVE (Image, City, Environment Laboratory), University of Strasbourg, 3 rue de l’Argonne, 67000 Strasbourg, France

**Keywords:** Assessment, Evaluation, Global North–Global South, Indicator framework, Practice, Urban green infrastructure

## Abstract

Indicator-based monitoring (IM) is crucial to support the uptake of Nature-based Solutions (NbS), to improve the knowledge of NbS benefits, and to promote evidence-based decision-making. Despite the growth of indicator frameworks to support practitioners in setting up IM processes for NbS, practitioners’ perspectives and experiences of IM application, especially in relation to NbS, have been underexplored. We address this knowledge gap through an exploratory study on practitioners’ decisions behind NbS-related IM through ten semi-structured interviews with experts from seven cities in the European (EU) and Latin American (LAC) region. LAC and EU practitioners use IM foremost to advocate the relevance and urgent need for improving urban sustainability. Novel compilations of eight applications of NbS-related IM in practice and three approaches to developing internally applied indicator frameworks were identified. More consideration of social indicators, technical feasibility, legitimacy, and temporal scales is needed for better and more inclusive NbS-related IM in practice.

**Supplementary Information:**

The online version contains supplementary material available at 10.1007/s13280-025-02174-0.

## Introduction

The urban local context is increasingly brought to the forefront of promoting and implementing Nature-based Solutions (NbS) (Wamsler et al. 2020; Adams et al. 2023). Building on concepts such as blue and green infrastructure, ecosystem-based approach, and ecosystem services (Dumitru et al. 2021), NbS are defined as existing and novel as “actions to protect, conserve, restore, sustainably use and manage … ecosystems which address social, economic, and environmental challenges effectively and adaptively, while simultaneously providing human well-being, ecosystem services, resilience, and biodiversity benefits’’ (UNEP 2022), which excludes actions not directly based or contributing to functioning ecosystems (Sowińska-Świerkosz and García 2022). However, the uptake of NbS in urban development is still hampered by different barriers, including lack of knowledge (Dorst et al. 2022; Collier et al. 2023) on the short- and long-term effectiveness of NbS; the environmental, economic, and social phenomena and processes underlying NbS-related challenges, such as climate change and biodiversity loss; the design, implementation, management, and governance processes supporting NbS; and society’s various interactions with NbS (Albert et al. 2019; Frantzeskaki et al. 2019; Rödl and Arlati 2022). Addressing these knowledge gaps requires robust and specific indicator-based monitoring (IM) (Sarabi et al. 2019; UNEP 2022).

In this paper, we refer to *IM* as the process of assessing, evaluating, and monitoring NbS and their impact through indicators at any given moment, from short to long periods (Haase et al. 2014; Dumitru et al. 2021). *Indicators* are parameters based on verifiable data that help to track data and communicate information about NbS, its benefits, and related phenomena and processes, such as climate change and demographics (Kumar et al. 2021). Although IM has been long addressed in sustainability research (Pinfield 1996; Huovila et al. 2019), only in the last years different assessment frameworks have emerged to support NbS-related IM (e.g., Albert et al. 2016; Maes et al. 2016; van Oudenhoven et al. 2018). Some of these have a specific focus, e.g., to assess climate adaptation (e.g., Calliari et al. 2019) or community governance (e.g., Muwafu et al. 2024), while others, such as the EU Handbook “Evaluating the impact of nature-based solutions”, provide comprehensive lists of indicators for different NbS-related challenges (Dumitru et al. 2021). Some studies focus on supporting practitioners in developing collaborative IM processes through step-by-step approaches (e.g., Rödl and Arlati 2022; van der Jagt et al. 2023). However, there is uncertainty about whether current indicators and assessment frameworks contribute to evidence-based decision-making (Berghöfer et al. 2016; van Oudenhoven et al. 2018).

Indicators and frameworks for IM, including those related to urban NbS, are mainly developed top-down by non-governmental actors with an academic background (Moreno Pires et al. 2014), focusing on credibility, accuracy, and completeness (van Oudenhoven et al. 2018; Carmen et al. 2020). Framework developers often overlook the practical realities and constraints of local practitioners (van der Jagt et al. 2023). This disconnect can lead to indicators, frameworks, and IM processes that are complex, irrelevant, and cost-intensive for local application by practitioners (Evans and Guariguata 2008; Berghöfer et al. 2016).

Nevertheless, due to the growing awareness of evidence-based decision-making, practitioners’ interest in conducting systematic IM has increased (Dumitru et al. 2020; Handmaker et al. 2021). IM allows them to gain first-hand information on the progress, benefits, and impacts of their own NbS projects and their city’s performances, to learn from their successes and failures, and to account for policy objectives (Sarabi et al. 2019; van der Jagt et al. 2023). However, due to the intricacies of environmental urban governance and limited resource availability, IM in practice tends to be pragmatic rather than systematic (McKenzie et al. 2014; Carmen et al. 2020) or might even be omitted altogether (Moreno Pires et al. 2014). So far, knowledge on measuring NbS impacts and, to a certain extent, their benefits is limited and has chiefly emerged from academia (Dumitru et al. 2020; Perosa et al. 2021).

Meanwhile, the learnings of practitioners and citizens from participating in IM processes and incorporating the knowledge into the co-designing, co-implementing, and co-managing of NbS have been overlooked in NbS research (Cvitanovic et al. 2016; Kabisch et al. 2016). Some studies address indicator selection in practice (Dobbs et al. 2019; Carmen et al. 2020), allowing the identification of biases toward monitoring specific aspects of NbS, such as regulating air quality, although IM frameworks should alongside environmental also include social and economic indicators (Kabisch et al. 2016; Dobbs et al. 2019). Occasionally, the uptake of academically developed indicator frameworks (Huovila et al. 2019) or the development of local indicator frameworks in practice is considered (Evans and Guariguata 2008). Most of these studies are reviews of indicators, indicator sets, or academic publications without considering NbS-related IM from a practitioner’s perspective.

Understanding IM-related urban governance practices from a practitioner’s perspective is essential in advancing IM in city practices (Turnhout et al. 2007). Recognizing practitioners’ motives for applying IM can promote it more effectively while understanding indicator and indicator framework selection, use, and development might help to make them more applicable and aligned with practitioners’ work routines (Berghöfer et al. 2016; van der Jagt et al. 2023). Yet, urban governance practices vary across cities and countries, affecting the relevance and feasibility of NbS indicators and monitoring regimes (Michalina et al. 2021). Studies on NbS-related IM show a bias toward European and North American cities (Chausson et al. 2020; Mercado et al. 2024), neglecting the importance of including a diversity of urban governance practices, in particular from the Global South (Adams et al. 2023). Despite their potential for NbS, cities from Latin American countries (LAC) face challenges concerning NbS implementation, such as high socio-economic disparities, weaker governance structures, knowledge fragmentation, and a lack of resources, capacities, and openness to participatory processes (Ryan and Bustos 2019; Breen et al. 2020; Pauleit et al. 2021). Such challenges are also likely to hinder NbS-related IM in city practices.

In this study, we take a cross-regional perspective and explore how NbS-related IM is applied in city administrations across EU and LAC cities. We aim to understand the motivations for conducting NbS-related IM and applying the resulting data and information in decision-making. In addition, we want to gain insights into how NbS-related IM is conducted, what is monitored in terms of indicators and indicator frameworks, and if the socio-cultural context influences the choice for an NbS-related IM procedure by juxtaposing cases in EU and LAC cities. For these reasons, we asked the following questions:

- Why do city practitioners apply NbS-related IM?
- How is IM of NbS applied in city administrations?
- What is being addressed in NbS-related IM: indicator frameworks, indicators, spatial and temporal scales?

## Materials and methods

This study, within the EU H2020 CONEXUS project framework, aimed to improve the knowledge of NbS, their benefits, and the processes supporting their uptake in ‘Life-Labs’ from seven in Europe (Barcelona (ES), Lisbon (PT) and Turin (IT)) and Latin America (Bogota (CO), Buenos Aires (AR), Santiago (CL), São Paulo (BR)) (see https://www.conexusnbs.com/). These cities are state or regional capitals with large administrations and experience tackling the challenge of sustainable urbanization and a vision to implement NbS. To delve deeper into the current collaborative processes of IM on NbS in practice (see van der Jagt et al. 2023) and on knowledge transfer through guidance material, such as handbooks and manuals, we conducted ten semi-structured interviews (Flick 2014). The interviewees include one academic and twelve city or regional government representatives with leading positions in planning and environmental departments across the Life-Labs with extensive work experience between 11 and 35 years in planning, mainstreaming, or monitoring environmental and greening policies and projects (see Table 1 for an overview of the interviews). Gender equality standards were applied for the interviewee selection, resulting in eight female and five male interviewees. However, within our sampling, gender played no significant role in defining and assessing IM within our sampling. They were identified through their involvement in the Life-Labs or recommended by local project partners for their expertise in the interview topics within their city’s context. The academic was included for his long-standing experience in IM in collaboration with the city and independently as a researcher and member of a citizen initiative. Interviewees were contacted by email introducing NbS, the related challenges (based on Dumitru et al. 2021), and the main interview topics and including an information sheet and consent form. In our communication with the participants, in line with the UNEP definition, we referred to the ability of NbS to restore natural ecosystems and to improve the quality of life in and around cities while mentioning the related concepts of ‘urban nature’ and ‘green infrastructure’ to avoid misunderstanding as NbS was still an emerging concept in LAC at the time of the interviews. Before the interview, all interviewees provided their written consent to record the interview and use the data. This study design and the handling of personal data within the study were approved by the Ethics Committee of the Technical University of Munich (approval no. 8/21S).

**Table 1:** Overview of interviewees with their identifier, affiliated city, affiliation, background discipline, and years of work experience within fields related to NbS. ^1^City abbreviation (capital letters) + number of interview + optional, in case of a group interview, an identifier for the individual interviewee (lower case letters), ^2^Approximation of the years of work experience in the field of NbS (e.g., built environment, nature conservation) based on interviews and public data

Identifier^1^	City	Organization	Background discipline	Years of work experience^2^
BC1a	Barcelona	Barcelona City Council—Municipal Institute of Parks and Gardens	Agricultural sciences	21
BC1b	Barcelona	Barcelona City Council—Municipal Institute of Parks and Gardens	Biology	35
BC1c	Barcelona	Barcelona Regional—Area of Environment, Energy and Data	Environmental sciences	24
BO2	Bogota	Pontificia Universidad Javeriana—School of Environmental and Rural Studies	Environmental sciences	21
BO3a	Bogota	City of Bogota—District Secretariat of Environment	Chemical Engineering	11
BO3b	Bogota	City of Bogota—District Secretariat of Environment	Environmental Engineering	12
BA4	Buenos Aires	National Secretariat for Territorial Development, Habitat and Housing	Law	14
BA5	Buenos Aires	Buenos Aires City Government, Environmental Protection Agency	Biology	35
LI6	Lisbon	Lisbon City Council, Municipal Directorate for Environment, Green Structure, Climate and Energy	Architecture and planning	18
SA7	Santiago	Regional Government of Metropolitan Santiago	Architecture and planning	20
SA8	Santiago	Ministry of Housing and Urban Development	Architecture and planning	15
SP9	São Paulo	City of São Paulo, Municipal Secretary of Green and the Environment	Geology	19
TO10	Torino	City of Turin, Environment Area	Environmental and planning studies	20

The interviews began with a brief introduction of the CONEXUS project and the aim of the study, offering a chance for clarifications regarding the provided information and interview topics, including the NbS concept. The first questions concerned general inquiries on local sustainability and governance challenges. These were followed by more in-depth discussions on IM and the knowledge transfer potential of guidance material (see Appendix S1 for the interview questions). This study focuses on the answers to the first questions of the interview related to the why, how, and what of IM.

To counter any multilingual challenges, persons with English, Portuguese, and Spanish proficiency were involved in the study. The interview questions were translated into Spanish and Portuguese to support interviewing in local languages. From March to July 2021, we interviewed the experts in the language of their choice, if possible, through online video conferencing applications (e.g., Google Meet, Zoom). The interviews—seven Spanish, two Portuguese, and one English—were recorded by audio recorders or directly in the video conferencing applications. The recordings were transcribed orthographically (Braun and Clarke 2013) with Descript (Descript), translated to English, if needed, with the DeepL tool (Kutylowski 2017), and anonymized by a co-author proficient in English, Portuguese, and Spanish, which offered a first quality control. Difficulties with translations were encountered for the Spanish interviews. Spanish-speaking interviews often spoke in long sentences, making it challenging to obtain clearly defined answers. In addition, they tend to end answers with a “¿no?” as a way of speaking, which might be interpreted by translation software as a negation, leading to changing the meaning of answers in the translations. For these reasons, the correctness and comprehensibility of the translations were checked for their correspondence with the original transcriptions and, if needed, adjusted. Awareness of these aspects was raised among the researchers who were not proficient in the original languages by collaborating with proficient colleagues.

We applied an explorative qualitative content analysis to analyze the transcribed interviews (Lamnek and Krell 2010). They were first screened to identify the relevant segments on IM, which were further divided into excerpts of one to ten sentences capturing an idea or experience related to IM into a table. Each excerpt was tagged with an identifier indicating the city abbreviation, interview number, in case of a group interview, the individual interviewee, and excerpt number (e.g., BC1c#34). The tagged excerpts addressing IM were deductively coded and categorized for the why, how, and what of IM based on the research and interview questions. In the next round, we applied inductive coding of the excerpts to identify patterns and themes within each category based on meaning and frequency (Braun and Clarke 2013), which formed the basis for descriptive summaries. The identifiers (e.g., BC1c#34) were used to track the source and frequency of summarized ideas and experiences. A co-author with Spanish and Portuguese language skills verified the interpretation of the summaries with the original transcripts and translations. The resulting summaries were included in the results section.

## Results

Below, the status quo, barriers, and potential of NbS-related IM in city administrations as perceived by the thirteen experts are described. We explore the relevance of IM for city administrations (‘why’), the different applications of IM and the approaches to using indicator frameworks for indicator selection (‘how’), and the practices of indicator selection and the role of scale in IM (‘what’). References to specific contexts in texts or through identifiers illustrate ideas and activities mentioned by the experts.

### Why IM is applied

The interviewees acknowledged the relevance of IM in increasing awareness (BA4, LI6) and understanding of NbS (BC1b, BO3a) and related topics, such as climate change (BO3a) and urban nature (BA4). They stated that IM aids informed decision-making processes (BC1b, BA4, BA5, SP9) in favor of more considerate choices for urban nature (BA4) by advocating NbS benefits (SA7) and getting evidence for promoting NbS to politicians. Data collected through IM can strengthen the arguments and legitimacy of NbS (BC1b, BA4, BA5, LI6, SA7, SP9), although changing mindsets can take years, as interviewee BA5 testimonies. Ideally, managers and politicians should make well-informed decisions (BC1b, SA7, SP9) based on scientific, reliable, and up-to-date information, which IM can provide (BA4). Access to IM data and information was seen as an important incentive for uptake (BC1a, BO3a, SP9) but not a guarantee (SA8).

Concerns were raised about sustaining NbS-related IM and informed decision-making in the long term in Buenos Aires (BA4), São Paulo (SP9), and Torino (TO10). Compared with challenges regarding housing, security, and quality of life, LAC interviewees indicated that NbS were not considered a priority (BO2, SA8). Moreover, across all LAC cities (BO2, BA4, SA7, SA8, SP9), a lack of informed decision-making was noted, which interviewee BA4 attributed to the lack of systematized and up-to-date IM; insufficient time to look at reliable information; no political willingness to conduct IM; and prioritizing intuition and social sensitivity over evidence. Interviewee BO2 indicated a lack of acceptance of IM outcomes, and interviewee SA7 confirmed the lack of political priority as:

*“the regional government is … concentrating on … security, emergencies, public order, rather than planning for the future” * (SA7, translated from Spanish).

These concerns regarding lack of informed decision-making also apply to civil society and private sector actors (BA, BO2, SA8). The demand for indicator information (BC1b) and pressure for positive change (LI6) by these actors seems stronger in EU cities, which also brings new challenges around additional workload and conflicts of interest (BC1b, LI6). Opportunities for civil society and private actors to take ownership of environmental issues also increased in Latin America (BO2), such as having IM data and information available. However, these actors still rarely apply IM outputs (BO3a, BA4) or use them to steer decision-making (BO2, SA8). Interviewee BO2 sketched the lack of informed decision-making by different actors as follows:

*“…the officials of municipal administrations who have to act and sometimes do so without much knowledge, and a citizenry that criticizes this very strongly without much knowledge either…” * (BO2, translated from Spanish).

On a positive note, Latin American interviewees indicated that activist groups (BA4), NGOs (BO2, SP9), and academia (BO3a, BA4) are considering or implementing NbS-related IM or developing IM frameworks (SA7).

### How IM is applied

In all seven city administrations, IM was conducted, with different departments conducting IM (BO2, TO10) and with three cities having specialized departments in environmental monitoring (BC1a, BO2, BO3a, SP9). For all European cities and Bogota, IM data and information were used as a basis for policy development (BC1b, BO2, BO3a, TO10) and for “*revision of plans and projects*” (LI6, translated from Portuguese). For São Paulo (SP9), an upward trend in IM use was reported, as the administration was setting up an interdepartmental monitoring system, although the lack of capacity and resources for setting up IM was a concern as indicated:

*“…until I train the team, have hardware, software, field monitoring, all this weakens the implementation” * (SP9, translated from Portuguese).

A decline in the use of IM was noted in Santiago (SA7) due to limited collaboration across sectors and regional ministries (SA7, SA8). Besides city administrations, NbS-related IM was considered or implemented by mayor offices (BO2), city councils (BO3), and other governmental institutions (BC1b, BO3a, BA4, BA5, SA7, TO10).

The interviewees provided broad descriptions of various ways IM was applied or ways they want IM to be conducted in their city. By analyzing these descriptions and their frequency through iterative refinement loops, we identified and formulated eight distinct IM applications indicating different purposes based on the interviewees’ descriptions (see Table 2). ‘Assessing conditions and developments of NbS, the built environment, and NbS-related processes’ was indicated for all cities, whereas ‘assessing NbS-supporting governance processes’ was the least frequently applied, with interviewees BC1a and SA8 explicitly mentioning not using the latter application.

*“We do not have any way of assessing this governance of urban nature and the impacts it has” * (SA8, translated from Spanish).

**Table 2:** Identified monitoring & indicator applications (applied and desired)

	Applications: assessing…	Mentioned by no. of interviewees	Mentioned by interviewees as applied	Mentioned by interviewees as desired
1	… **conditions and developments of NbS, the built environment, and NbS-related** environmental, social, or economic **processes**, e.g., areas of green space and local climate conditions, as a source of information for NbS implementation plans and policies	11	BC1b, BC1c, BO2, BO3a, BA4, BA5, LI6, SA7, SA8, SP9, TO10	
2	… the **impact of NbS and NbS-related projects** through ex post evaluation, e.g., through ecosystem services	7	BC1a, BA4, SA7, SA8, SP9, TO10	BC1a, BO2, BA4, SA7, SA8, SP9
3	… the **progress of NbS projects and plans** through executing actions and tasks, e.g., how much of the planned activities have been executed	7	BC1a, BC1b, BO3a, BA5, LI6, SA8, TO10	
4	…**the performance of NbS based on comparisons** with other interventions, cities, or regions, often through ex ante or cost–benefit analysis	6	BC1b, SA7, SP9	BC1a, BO2, BA4, SA7, SP9
5	… the **progress made toward environmental objectives** defined in a city’s policies, e.g., an x amount of green space per capita by 2030	5	BC1a, BC1c, SA8, TO10	BC1a, SP9
6	… the impact or **effectiveness** of NbS-supporting instruments and policies, e.g., whether they have the intended effect	4	TO10	BC1a, BO3a, BA4, TO10
7	… whether environmental **criteria** for certification **or legal threshold values** are being met	2	LI6, TO10	
8	… **NbS-supporting governance processes**, e.g., whether and how well NbS-supporting governance structures, procedures, and interactions are conducted or implemented	1	TO10	

The text in bold indicates the name of the applications, as is used in the full text.

Most cities mentioned three or five types of IM applications being implemented. Torino even utilized seven different ones, showing that one city administration can apply IM for several purposes. Applications can also build upon each other. For example, interviewee SA7 pointed out that they assessed the status quo and the performance of interventions, which could serve as a baseline for an ex-post performance evaluation of projects by higher-scale authorities, for example: *“the Ministry of Social Development … conducts ex-post evaluations*” (SA7, translated from Spanish). This demonstrates that IM applications can be implemented through a network of different organizations. Interviewees expressed a wish to conduct specific IM applications or, if they had already adopted the application, use it more or better (BA4, SA7, SA8, SP9, TO10). An application especially favored by Latin American interviewees is evaluating the impact of NbS (BC1a, BO2, BA4, SA7, SA8, SP9) and comparing the performance of different NbS (BC1a, BO2, BA4, SA7, SP9). The latter allows for an improved understanding of the costs and benefits of NbS (BC1b, BO2, SA7), benefitting their case for NbS (BA4, SP9).

### How indicator frameworks are applied

Three distinct approaches emerged during the inductive coding, describing how the interviewees used existing indicator frameworks to develop internally applied indicator frameworks (see Table 3). Each of the three approaches was defined based on the interviews, and a name was provided to characterize each approach. In DIY, indicators are created in-house based on local objectives, NbS implementation needs and possibilities, and local capacities and resources, as exemplified by Torino’s Strategic Green Infrastructure Plan (TO10). Mix & Match involves compiling a framework by selecting indicators from various sources: internally managed databases (BC1a) or external frameworks, like from the Forest Stewardship Council (FSC) (TO10), the United Nations Economic Commission for Latin America and the Caribbean (ECLAC) (BO3a), the Intergovernmental Panel on Climate Change (IPCC), the Sustainable Development Goals (SDG) (BA5, SP9). In Carbon Copy, existing frameworks are adopted, often to meet national (BA5) or international (LI6) legislative targets, with examples from C40 cities partnership^1^ , the CDP’s climate reporting platform^2^ (LI6, SP9), or competitions, such as the European Green Capital European Commission, Directorate-General Environment, Directorate C - Zero Pollution, ENV.C.3 - Clean Air & Urban Policy. n.d. European Green Capital and Leaf Awards 2026. Call for Applications. Rules of Contest. Luxembourg: European Commission. (LI6). These approaches can exist alongside each other in the same organization and can converge with one another.

**Table 3:** Practitioners’ approaches to the development of internally applied indicator framework

#	Approach	Description
1	DIY (do-It-yourself)	The indicator framework and the indicators are developed in-house based on local objectives
2	Mix & match	The indicator framework is developed by selecting indicators from pre-existing different sources: internal databases, external indicator frameworks and indexes
3	Carbon copy	The indicator framework is one-on-one adopted from external predetermined indicator frameworks without any adjustments

As interviewee SA7 mentioned, effective indicator frameworks should encompass environmental, social, and economic dimensions and allow for understanding the interdependencies between different indicators. According to interviewees BC1b and BA5, commonly applied frameworks support comparison between different cities and regions and across scales (locally, regionally, or globally), which is why in Torino:

*“[i]n general, frameworks are adopted that are coherent with the national strategy as it allows comparison across different cities and territories” * (TO10).

However, comparisons are only meaningful between cities and regions with similar characteristics. For example, in terms of population, budget, and management, it makes more sense for Barcelona to compare itself with other world cities than smaller cities in the region (BC1a). Challenges are a lack of relevant national frameworks related to NbS or the urban scale, as mentioned for Bogota (BO2), Buenos Aires (BA4), and Torino (TO10), as well as the competition of NbS frameworks with indicator frameworks for other sectors, such as transport (BA5).

### What IM is addressing

The interviews revealed that cities like Barcelona and Bogota have extensive lists of indicators spanning different themes (BC1b, BO2, BO3a) and indicator types (BC1a, BC1c), both simple and in-depth (BO2). Indicators directly referring to NbS were lacking or insufficient according to the interviewees BO2, BA4, LI6, and SA7, or as described for Barcelona:

*“From the point of view of evaluation, we do not have indicators of urban nature…They are in a framework; the only thing is that they are not properly classified as NbS indicators” * (BC1, translated from Spanish).

Instead, interviewees mentioned indicators related to green space management (BC1c, BC1a, BC1b, BO3a, BO3b, LI6, SA7, SA8), ecosystem services (BC1a, TO10), biodiversity (BC1a, BC1c, BC1b, BO3a, LI6), climate change (BC1a, BO3a, BA4, TO10), air quality (BO3a, BA4, LI6), water management (BC1c, BO3a, LI6), soil (BO3a, BA4, LI6, TO10), waste (BC1c, LI6), health (BA4, SP9) and sustainable production (BC1c, TO10).

Alongside these environmental indicators, IM should include social, economic, and governance indicators. Interviewee BA4 stated that including social and economic indicators is important to find adequate solutions:

*“…those who are more exposed to the degradation of nature are those with fewer resources. So, it is important to weigh up the environmental factor and the impact it has on these communities” * (BA4, translated from Spanish).

Yet, such indicators were less frequently or not considered at all. European interviewees BC1a and TO10 acknowledged that they lack the indicators and skills to assess social issues, such as citizen participation and well-being. Within Latin American cities, social aspects of NbS were being addressed. In Santiago, indicators related to welfare and indigenous issues were developed (SA7, SA8); São Paulo (SP9) assessed indicators on education; and Bogota assessed participation and citizen perception (BO2, BO3a). Interviewees BC1a, BC1c, and BA4 expressed an intention to use indicators on citizen participation, park use, and perceptions of the health and biodiversity impacts of green interventions.

Selection criteria for individual indicators were mainly provided by Latin American interviewees, referring to the policy relevance of themes such as environmental justice (BC1a, BO3a, SA8, SP9). Other considerations for indicator selection include legislative and technical feasibility (BO3a, SP9), e.g., available expertise (SA8); data availability (BO3a); applicability over extended time periods (BO3a); and adaptability to the local context (BA5).

As acknowledged by interviewee BC1a, long-term studies are essential to look beyond intermediate effects, particularly for health and well-being. In general, the city administrations focus on short-term, one-off assessments. Although long-term studies of NbS were mentioned for Barcelona (BC1b), Bogota (BO2), and São Paulo (SP9), where they “*created a set of indicators that will be monitored for the next 10 years*” (SP9, translated from Portuguese). Consideration of temporal scales is important, explained interviewee BO2; depending on the indicator, changes are assessed annually or monthly (BO3a). Moreover, interviewee BC1a indicated that indicators should be applied before and after plan implementation, as well as during the management phase. Considering spatial scales also received attention in the interviews (BO3b, BA5). Cities applied indicators at different spatial scales: neighborhood and district level (BC1a, SA8), large urban projects (BC1a, BC1c, SA7), or local NbS projects (BA4, SA8). Interviewee BO2 mentioned that, in Bogota, IM is conducted at the city and district scales, ensuring disaggregated information for the local decision-making level (BC1a, BO3b). Data on other spatial scales, like the regional scale, is sometimes assessed by other departments or institutions, like in the examples of Barcelona (BC1b), Buenos Aires (BA5), and Santiago (SA7, SA8). However, indicators might not be easily transferred (BA5) if their assessment is only applicable on larger scales (SA7) or they are too locally specific, as was the case for Buenos Aires:

*“…they are specific indicators for specific actions, I don’t know how much use they can be to another government…”* (BA5, translated from Spanish).

Regardless of the extent of IM, various interviewees, especially from Latin America, highlighted a need to implement a more systematic approach to IM (BO2; BA4, SA7, SA8, SP9, TO10).

## Discussion

### Use of IM data and information for informed decision-making on NbS

We found that practitioners in EU and LAC cities consider IM to enhance knowledge on NbS, supporting evidence-based decision-making and policy development, which aligns with the scientific and policy discourse on this topic (Newman et al. 2017; Collier et al. 2023). EU interviewees particularly noted IM as a basis for policies and plans. Although IM is conducted and considered by all city administrations and other public actors, cities with large administrations, like Barcelona, Bogota, and São Paulo, have specialized departments for environmental IM. In contrast, smaller cities or those with more fragmented governance structures, like Santiago, tend to lack the resources for specialized IM units. In addition, civil society actors engaged in IM in LAC cities, sometimes in collaboration with public actors (van Lierop et al. 2024). However, it is unclear whether it is a response to weak governance structures or part of the region’s long-standing tradition of bottom-up initiatives (Breen et al. 2020).

LAC interviewees indicated that the availability of IM data and information does not guarantee evidence-based decision-making. Practitioners might lack the capacity to adequately conduct IM or interpret its outcomes due to the lack of systematized IM or time. Organizational issues, such as poor leadership or power disparities, can further hinder the use of IM and its outcomes in evidence-based decision-making (Newman et al. 2017; Frantzeskaki et al. 2019). In line with Moreno Pires (2014), the interviewees gave a lack of political will, dismissal of IM outcomes, and prioritization of other factors over evidence as significant hurdles. These barriers underline that decisions are influenced by more than information alone but also by cognitive, emotional, and relational factors (Wamsler et al. 2020), which could explain a more conceptual, strategic, or even political use of IM data and information (Turnhout et al. 2007; McKenzie et al. 2014). Like Handmaker et al. (2021), the experts in the EU and LAC cities stressed the role of IM in communicating, promoting, and convincing others about NbS and their benefits. In contrast, studies on IM often underline the direct instrumental use of resulting data and information to support rational decision-making (McKenzie et al. 2014).

Understanding the governance enablers and the role of knowledge in the governance of IM can facilitate knowledge exchange and uptake by different actors to support evidence-based decision-making. Insufficient knowledge uptake and application are not limited to public decision-makers but extend to private actors (Reed et al. 2006), with the latter insufficiently being engaged, according to LAC interviewees. Selecting relevant indicators, producing purposeful data, and communicating information to the public can motivate citizen and community engagement (Agol et al. 2014; Cvitanovic et al. 2016). Yet, it is vital to stay vigilant to safeguard monitoring data from misuse for political reasons (Farrell et al. 2019).

### Applying NbS-related IM

We identified eight ways (i.e., ‘applications’) in which IM was utilized to assess or evaluate NbS or NbS-related phenomena or processes. Previous studies mention IM applications (e.g., Dumitru et al. 2021; Kumar et al. 2021), yet the few providing typologies of IM applications focus on specific domains. Rödl and Arlati (2022) concentrated on NbS design, and Helming et al. (2008) on environmental policies. These typologies did not consider ‘assessing whether criteria or legal threshold values are met’ or ‘assessing NbS-supporting governance processes’, which are the applications uncovered in this study. The interviewees reported ‘assessing the process of NbS projects and plans’ frequently, yet it is less included in other typologies (e.g., Fürst and Scholles 2008). Helming et al. (2008) and Rödl and Arlati (2022) aligned their typologies to process phases of policy-making or NbS implementation, yet such alignment could not be identified from the interviews.

The three most frequently employed ways of applying IM were: ‘assessing conditions and developments of NbS, the built environment, and NbS-related processes’ (in 7 cities), ‘assessing the progress of NbS projects and plans’ (in 6 cities), and ‘assessing the impact of NbS and NbS-related projects’ (in 6 cities). Within EU cities, a larger variety of IM applications were employed, whereas LAC interviewees expressed a desire for more assessment of NbS impacts and comparison of NbS performance. The preference for the latter compared with analyzing governance processes supporting NbS is in line with the literature (van der Jagt et al. 2023; van Lierop et al. 2024). These differences show that not every practitioner may be familiar with or value IM applications equally. Yet, being familiar with the different purposes of IM could help select the most suitable approach to collect the required data and information.

We determined that IM applications can build on each other, even if they fulfill different purposes and are conducted by different actors. When processes and impacts are monitored, linkages between both can be identified (Evans and Guariguata 2008), such as whether internal decisions or external factors define progress (Dumitru et al. 2020). Collaboration among stakeholders or between institutions can support implementing a broader range of IM applications to gain a more holistic understanding of NbS implementation and establishing baselines for future IM on impacts and processes (Reed et al. 2006), crucial in contexts with institutional fragmentation like in LAC cities (Ryan and Bustos 2019; Breen et al. 2020). The challenge lies in coordinating collaboration on IM among different stakeholders and aligning varying time frames, e.g., long-term governmental monitoring combined with short-term assessment of research projects or political cycles (Agol et al. 2014).

### Combining the three approaches to local indicator framework development

To our knowledge, this paper is among the first to explore practitioners’ application of existing indicator frameworks to develop local indicator frameworks for NbS. Past studies have mainly focused on the selection of indicators and targeted performances (Carmen et al. 2020; Pugliese et al. 2022), applying standardized indicator frameworks in specific contexts (Moreno Pires et al. 2014), or assessing the indicator frameworks themselves (Michalina et al. 2021). We identified three approaches for developing local indicator frameworks: DIY, Mix & Match, and Carbon Copy (see Table 3). The latter two showed local governments using pre-existing indicator frameworks, although mainly from global organizations. This preference could stem from city administrations’ connection to these organizations, users’ familiarity with the frameworks and their executive organizations, or their availability, relevance, and credibility (Pires et al. 2020; Handmaker et al. 2021). In line with Moreno Pires et al. (2014), interviews from EU and LAC cities indicated a lack of relevant national or local NbS frameworks.

Adopting predetermined generic indicator frameworks, as in the Carbon Copy approach, can support the systematic assessment of environmental, social, and economic indicators (Frantzeskaki et al. 2019; Sarabi et al. 2019). Using such generic frameworks across cities allows for gaining insight into the scale of current global changes, such as climate change and biodiversity loss, and their global, regional, and local implications (Robinson 2016) as well as comparisons (Moreno Pires et al. 2014; Huovila et al. 2019). Comparisons allow benchmarking one’s city with other cities (Kitchin et al. 2015). Yet, comparing a city such as Barcelona with other cities may be only relevant or fair if there are similarities in budgets, history of greening, climate, or available knowledge (Kitchin et al. 2015) or if normalization or tracking the percentage of change can be applied (Dobbs et al. 2011). To ensure systematic assessment, generic indicator frameworks are often comprehensive and complex, which makes them challenging to implement in contexts with different expertise and low capacity, including for citizen science purposes (Evans and Guariguata 2008). Generic indicator frameworks can also be less adaptable to local conditions, issues, and needs (Dumitru et al. 2020). As specified for Torino, the benefit of the first approach is its relevance for the local context, as indicators are specifically developed to match local objectives. However, the “Mix & Match” approach was the most frequently applied, which aligns with the preferences for pragmatism found by Carmen et al. (2020).

To combine the strengths of all three approaches, one could work with a generic core set of indicators across environmental, social, economic, and governance dimensions (Michalina et al. 2021); for example, from C40 or national frameworks, and a supplementary flexible set of indicators developed in-house through an inclusive, collaborative process to match local policies, needs, and NbS (van der Jagt et al. 2023). The core set would assure comparability and an integrative approach, while the supplementary set allows for assessing context-specific objectives and processes (Dumitru et al. 2020; Michalina et al. 2021). To ensure a feasible and continuous IM process, the core indicator set should be fixed and easy to understand, measure, and communicate (Evans and Guariguata 2008; Agol et al. 2014). The supplementary indicator set can contain more complex indicators catering to the evolving needs and capacities of involved partners.

### Indicators, selection criteria, and scales can be bettered

Selection criteria for indicators were mainly mentioned by LAC interviewees. Based on van Oudenhoven et al. (2018), none of the criteria referred to legitimacy or credibility but focused on saliency or feasibility. In LAC, there could be less reliance on frameworks, while the indicated criteria also relate to local needs, political agendas, or restrictions to IM uptake. The saliency criteria refer to the relevance of information to policy needs, transferability to local context, and applicability over time, while feasibility criteria are linked to legislation, technical issues, and availability of expertise and data. Van Oudenhoven et al. (2018) did not cover legal and technical feasibility, but other studies did (e.g., Agol et al. 2014; Berghöfer et al. 2016). Feasibility is an important criterion for individual indicators to ensure measurement and successful IM (Carmen et al. 2020). Van der Jagt et al. (2023) suggest applying legitimacy and relevance to the indicator portfolio as a whole. Not each indicator must be relevant or legitimate to the policy and information needs of all stakeholders; as long as there are indicators included that address the stakeholder needs, including those of marginalized groups, and allow assessment of underrepresented issues, such as environmental justice (van der Jagt et al. 2023). However, it is unclear from the interviews whether these selection criteria were applied at the aggregate level of the portfolio or the indicator level.

The practitioners showed a bias toward using ecological and environmental indicators, overlooking the multidisciplinary impacts of NbS, which include social, economic, and governance dimensions. This discrepancy has been addressed before in different contexts (Dobbs et al. 2019; Dumitru et al. 2021; Wickenberg et al. 2021). Yet, the transdisciplinary nature of NbS requires knowledge from natural and social sciences (Frantzeskaki et al. 2019), such as addressing environmental justice issues (Cousins 2024). Although the importance of assessing social NbS impacts was recognized in EU and LAC cities, LAC cities are more inclined to include social indicators compared to their European counterparts, possibly due to a higher presence of economically disadvantaged communities, emphasizing the relevance of social next to environmental indicators (Portugal Del Pino et al. 2020; Wild et al. 2024). Economic indicators were notably absent in the interviews despite the known financial barriers to NbS implementation (Droste et al. 2017; Sarabi et al. 2019) and the requests for more and improved cost-benefits evaluations (van Lierop et al. 2024; Wild et al. 2024).

Defining temporal and spatial scales of indicators is relevant for IM (Haase et al. 2014), as they can significantly affect data availability, required monitoring resources, and, potentially, decision-making (Kumar et al. 2021; Odongo et al. 2022). While interviewees mentioned IM on different spatial scales, the consideration of cross-scale interactions, which are relevant for understanding NbS impacts, remained unclear. NbS impacts might not be measurable on a micro-scale, but their cumulative impact can be significant at a meso-scale (Kumar et al. 2021). NbS monitoring should also consider short-term to long-term impacts. However, most IM studies neglect the NbS lifecycle, including the management phases (Odongo et al. 2022). Consideration of temporal scales is limited despite being key in assessing NbS’ full potential over time, especially for benefits linked to social cohesion and health (Haase et al. 2014; Odongo et al. 2022). Long-term studies are relevant to better understand NbS development, use over time, and management (Dumitru et al. 2020), and references to such studies were made. To further uptake spatial and temporal scales in IM, van Oudenhoven et al. (2018) propose prioritizing them in indicator selection.

### Reflection on interviews and interviewees

Exploratory studies with a limited sample, such as this one, cannot provide a complete overview of all aspects, e.g., of all NbS indicators used in the studied cities. This also means that when topics were not indicated for a city, this does not automatically imply that the topic was not considered. The IM application ‘assessing whether criteria or legal threshold values are met’ was linked for Lisbon and Torino with European legislation, which makes it likely Barcelona also employs IM for the same purpose. The individual interviewees’ backgrounds, expertise, and ongoing activities can also explain differences. Interviewee BC1b’s work focuses on biodiversity, while BA5’s relates to climate change. These topics come then more to the fore in the interviewees. Answers to IM might also be more or less extensive based on IM’s role in the interviewees’ daily tasks. More affinity and detailed knowledge of IM also seemed to come from interviewees with a background in natural or environmental sciences, as IM was more likely to be part of their curriculum.

Due to the novelty of the NbS concept, as indicated by three LAC interviewees, actions and indicators may not be explicitly labeled as related to NbS (European Commission. Directorate-General for Research and Innovation. 2024; van Lierop et al. 2024), which could explain the noted lack of NbS-specific indicators. Nevertheless, all interviewees were familiar with the concept of NbS and linked it most frequently to water management, climate resilience, and air pollution. NbS mainly were related to green public spaces, ranging from trees to parks, with a stronger focus on creating new solutions than conservation (Gantioler et al. 2022). In line with the UNEP definition, biodiversity and nature were considered essential aspects of NbS. However, using "urban nature" in the interviews might partially account for this finding.

## Conclusions and recommendations

This exploratory study provided new insights into how, why, and on what practitioners conduct IM in the seven local government settings from EU and LAC countries. Practitioners in both regions considered IM relevant for evidence-based decision-making. However, even if IM data and information are available, IM is still hampered by various barriers, as articulated by the LAC interviewees. Practitioners more frequently use IM to advocate the benefits of NbS. This political role underscores the need for better communication and implementation of IM data and information while at the same time being aware of political misuse. In this regard, decision-makers’ perspectives on IM could provide vital insights.

Using IM data and information as a basis for policies seems more common in EU cities, while in LAC cities, other actors, such as universities and NGOs, also consider and conduct IM. An overview of the different actors involved in IM or using IM data and information in a city and their motives can enhance the alignment of IM studies across spatial and temporal scales. In LAC cities, involving a wide range of actors is crucial due to limited state capacities and fragmented knowledge. A network of actors can also support using the eight identified IM applications, each serving a specific purpose and complementing one another to provide a more holistic picture of NbS implementation. The IM applications are recognized with varying degrees in theory and practice of EU and LAC cities. Further research is needed on how, when, and under which conditions the different applications are applied in practice and their perils and potentials.

We outlined three approaches to developing local indicator frameworks: DIY, Mix & Match, and Carbon Copy. The latter two utilize existing indicator frameworks, mainly of global organizations, whereas specific NbS-related frameworks were not mentioned. We propose combining the strengths of the three approaches by using a fixed integrative core set of indicators supplemented by locally relevant indicators—either selected or developed. Similarly, pragmatic choices for salient and feasible indicator selection criteria appeared to be common practice in LAC, with technical feasibility requiring better consideration in IM. The same accounts for legitimacy, which was not mentioned as a selection criterion despite its relevance for inclusive processes. In addition, we call for better inclusion of spatial and temporal scales in IM, which is currently still limited.

In line with previous studies, environmental indicators were more frequently applied in both regions, while LAC cities are leading in monitoring the social outcomes of NbS. Economic indicators were overlooked in both regions despite frequent calls for cost-benefit analyses by LAC practitioners. Future studies should complement expert interviews with analyses of indicator sets in local monitoring plans to deepen insights into indicator themes and types (e.g., Huovila et al. 2019; Carmen et al. 2020). As the study focused on LAC and EU cities with large city administrations, we recommend expanding research on why, how, and on what IM is conducted in smaller city administrations and other global regions, such as sub-Saharan Africa, Southeast Asia, and Eastern Europe.

## Supplementary Information

Below is the link to the electronic supplementary material.

Supplementary file1 (PDF 83 kb)

## Footnotes

1 (www.c40.org).

2 (www.cdp.net).
