# Supplementary material for: The why, how, and what of indicator-based monitoring of nature-based solutions: Perspectives from EU and LAC city practitioners
Source: Ambio. 2025 Apr 22;54(10):1621–34. doi: 10.1007/s13280-025-02174-0 (PMC12405076; doi:10.1007/s13280-025-02174-0)
Supplement: Supplementary file 1 — Supplementary file1 (PDF 83 kb) [file 13280_2025_2174_MOESM1_ESM.pdf]

Supplementary Information

The Why, How, and What of Indicator-based Monitoring of Nature-based Solutions: Perspectives from EU and LAC City Practitioners

Martina van Lierop[a], Cynnamon Dobbs[b], Alexander van der Jagt[c], Andrea Skiba[d], Camila Flores[e], Giuliano Maselli Locosselli[f], Denise Duarte[g], Aude Zingraff-Hamed[hi], Stephan Pauleit[j]

[a] Research and Teaching Associate, Chair for Strategic Landscape Planning and Management, School of Life Sciences Weihenstephan, Technical University of Munich, Emil-Ramann-Straße 6, 85354 Freising, Germany. Email address: martina.van-lierop@tum.de

[b] Assistant Professor, Centro de Estudios Territoriales, Universidad de los Andres Chile, Monsenor Alvaro de Portillo 12455, Las Condes, Santiago Chile, Chile. Email address: cdobbs@uandes.cl

[c] Assistant Professor, Institute for Place, Environment and Society, School of Energy, Geoscience, Infrastructure and Society, William Arrol Building, Heriot-Watt University, Edinburgh EH14 4AS, United Kingdom. Email address: s.van_der_jagt@hw.ac.uk

[d] Research Associate, Chair for Strategic Landscape Planning and Management, School of Life Sciences Weihenstephan, Technical University of Munich, Emil-Ramann-Straße 6, 85354 Freising, Germany. Email address: andreaskiba@gmail.com

[e] Research Assistant, Chair for Strategic Landscape Planning and Management, School of Life Sciences Weihenstephan, Technical University of Munich, Emil-Ramann-Straße 6, 85354 Freising, Germany. Email address: bycamilaflores@gmail.com

[f] Assistant Professor, Center for Nuclear Energy in Agriculture, University of São Paulo, Av. Centenário, 303 - São Dimas, CEP: 13416-000 - Piracicaba (SP) – Brazil. Email address: locosselli@cena.usp.br

[g] Full Professor, University of São Paulo, Faculty of Architecture, Urbanism and Design, Rua do Lago 876 Cidade Universitária, 05508-080 São Paulo-SP, Brazil. Email address: dhduarte@me.com

[h] Assistant Professor, ENGEES National Institute for Water and Environmental Engineering, 1 cour des cigarières, CS 61039, 67070, Strasbourg, France. Email address: aude.zingraff-hamed@engees.unistra.fr

[i] Assistant Professor, University of Strasbourg, CNRS, ENGEES, UMR 7362 LIVE (Image, City, Environment Laboratory), 3 rue de l'Argonne, 67000, Strasbourg, France

[j] Full Professor, Chair for Strategic Landscape Planning and Management, School of Life Sciences Weihenstephan, Technical University of Munich, Emil-Ramann-Straße 6, 85354 Freising, Germany. Email address: pauleit@tum.de

Corresponding author

* Corresponding author: Martina van Lierop, Email address: martina.van-lierop@tum.de; Telephone: +49 8161 714661

Appendix S1 Questionnaire for interviews

Ahead of the interview
Briefly introduce yourself. Briefly describe the CONEXUS project and the aim of this specific study. Check if the interviewee received and read the information sheet. Make sure they have read through and signed the consent form.

Record the interview, using a voice recorder or the video conferencing software you are using.

Main sustainability challenges

- What are the main sustainability challenges[1] in the city, and what is the potential role of NBS in addressing these?

Governance challenges

- Which departments and/or sections in the municipality are directly engaged in the development and maintenance of nature-based solutions and how have you organised your working relationship with (other) relevant departments? Are there any actors or departments missing?
- What are the main governance challenges around steering the actions of actors relevant to urban nature development, and how do you aim to address these?

Assessment
Relevance of assessment

- How important is assessment of urban nature to you? What are the main arguments for doing it (e.g. political influence, justification to co-investors, reporting progress against sustainable development goals)?

Assessment process

- In what ways are you assessing the governance of urban nature and the impacts of urban nature? Where and at which scale(s) do you measure?
- What are the challenges?

Use of assessment frameworks and indicators

- Which are the assessment framework(s) and/or indicators that you are using?
- Why did you select these particular assessment frameworks and/or indicators and not others? What have been the key arguments for selecting these?
- Are you familiar with other types of assessment frameworks or indicators?

---

[1] We are interested in the following broad categories of sustainability challenges:
1. Climate Resilience
2. Water Management
3. Natural and Climate Hazards
4. Green Space Management
5. Biodiversity
6. Air Quality
7. Place Regeneration
8. Knowledge and Social Capacity Building for Sustainable Urban Transformation
9. Participatory Planning and Governance
10. Social Justice and Social Cohesion
11. Health and Well-being
12. New Economic Opportunities and Green Jobs

- Do you perceive any opportunities for the CONEXUS assessment framework to complement current assessment approaches for urban nature in the city? In what ways might it do so?

Actors involved in assessment

- Who is conducting the assessment within your organisation?
- Are urban stakeholders from outside the city administration involved in indicator selection, data collection or analysis?
- What are the advantages and disadvantages of having external actors involved?

Use and users of assessment outcomes

- What types of actors (e.g. policy makers, politicians) are using the outcomes and for which purposes?
- Who would you like to use the assessment outcomes? Why do they not use them?

NbS guidance material

Selection and use of guidance

- Is there any specific guidance (e.g. handbooks, manuals, toolboxes, guidelines, websites), which you consult now or in the past for the assessment, planning or governance of NbS (or related concepts)?
- How often do you use this guidance and under which circumstances? And why these? How do these NbS guidance support you?
- Which criteria are you using to evaluate the relevance of a guidance (e.g. whether the information is easy accessible or understandable)?
- Are you aware of colleagues using this or other types of guidance? And how does the guidance support them?
- Do you think that the guides, manuals and tools are used by professionals?

Guidance needs

- Do you think there are specific topics insufficiently addressed in NbS guidance?
- Do you think there is certain guidance missing?

Recommendations for guidance

- If you compare a good example of guidance with a bad example of guidance, what would be the differences?
- How would you improve current NbS guidance?
- What recommendations would you give us for developing the CONEXUS ecosystem of guidelines?

Suitable formats for guidance

- What are according to you suitable formats (e.g. online or print, websites, booklets, videos, online courses, blogs) to share/transfer information for guidance on NbS (or related concepts)
